# Disability Acceptance as a Key Protective Factor Against Depression: Evidence from Korea’s National PSED (Wave 2)

**DOI:** 10.3390/medicina62020301

**Published:** 2026-02-02

**Authors:** Yoon Kyoung Jeong, Gyeong Min Lee, Jae-Hyun Kim

**Affiliations:** 1 Department of Health Administration, College of Health Science, Dankook University, Cheonan-si 31116, Chungcheongnam-do, Republic of Korea; sarah041123@naver.com; 2 Dankook Institute for Health & Medical Policy, Dankook University, Cheonan-si 31116, Chungcheongnam-do, Republic of Korea; lgm910212@yonsei.ac.kr

**Keywords:** disability acceptance, depression, people with disabilities, mental health disparities, population-based study

## Abstract

*Background and Objectives*: Depression is a significant concern in Korea, where people with disabilities show a 3.7 times higher prevalence than the non-disabled. While disability acceptance is linked to positive outcomes like self-esteem, its direct association with depression is underexplored. This study examined the relationship between disability acceptance and depressive symptoms in this population. *Materials and Methods*: This study used data from the second wave of Korea’s Panel Survey of Employment for the Disabled (PSED). The analysis included 4030 registered individuals with disabilities. Logistic regression was used to estimate the association between disability acceptance and depressive symptoms, adjusting for sociodemographic and health-related factors. *Results*: The overall prevalence of depressive symptoms was 18%. A strong dose–response relationship was observed: depression rates were 59.7% in the lowest disability acceptance group versus 5.1% in the highest. After adjustments, the lowest acceptance group was over 11 times more likely to experience depression than the highest group (OR = 11.35, 95% CI = 5.86–22.00, *p* < 0.0001). *Conclusions*: Lower disability acceptance was significantly associated with a higher risk of depression, indicating it is a key protective factor for mental health. Interventions should therefore focus on enhancing disability acceptance through counseling, social support, stigma reduction, and policies promoting social integration.

## 1. Introduction

Depression has emerged as a significant mental health issue in South Korea, severely diminishing the quality of life for individuals. According to a paper comparing the Korean Epidemiologic Catchment Area Study (KECAS), which surveyed the prevalence and risk factors of mental illness in Korean adults, and the National Comorbidity Survey (NCS), a representative national survey on the prevalence and comorbidity of mental illness in American adults, the prevalence of Major Depressive Disorder (MDD) in Korea is 2–4%, lower than the approximately 10% in the United States [1]. However, despite this low prevalence, South Korea’s suicide rate is the highest among OECD countries [2]. This suggests that the severity of depression cannot be assessed by prevalence rates alone. Furthermore, while Koreans often report somatic symptoms such as fatigue or difficulty concentrating, they are relatively less likely to express emotional states like depressive moods or suicidal ideation, creating a risk of underdiagnosis [1]. Therefore, the assessment of depression must go beyond surface-level emotional expression to consider both physical and psychological symptoms.

Depression is not merely an individual emotional problem but a major public health issue that can lead to impaired social functioning, interpersonal difficulties, and, in severe cases, suicide [3]. Particularly since the COVID-19 pandemic, issues such as social isolation and economic instability due to job loss have contributed to a rising trend in the prevalence of depression, underscoring the importance of early detection and intervention [4]. The World Health Organization (WHO) also classifies depression as a major disease that significantly contributes to Disability-Adjusted Life Years (DALY), indicating that South Korea is no exception to this global trend [5].

While the experience of depression is high even in the general adult population, the problem is even more severe among individuals with disabilities. Some studies report that the prevalence of depression among people with disabilities is 3.7 times higher than in those without disabilities, a result understood to be a complex interplay of psychosocial factors, not just physical health issues [6]. The mental health problems of people with disabilities are closely linked to various structural factors, including not only physical limitations but also social stigma, discrimination, social isolation, economic hardship, and poor living conditions [7]. The constraints imposed by a disability create persistent limitations in areas such as career choice, educational opportunities, and the formation of social relationships, which in turn lower overall life satisfaction and deepen feelings of emotional isolation [8]. Some research also suggests that depressive symptoms are more pronounced in individuals with disabilities who have lower levels of social participation [9]. Furthermore, in addition to these external stressors, an individual’s mental health can be significantly influenced by how they perceive and accept their own disability. That is, the degree of disability acceptance is gaining attention as a critical internal factor affecting the psychological well-being of individuals with disabilities [10]. Disability acceptance refers to the process by which an individual incorporates their disability into their self-identity and comes to believe that the disability does not diminish their life’s value or meaning [11]. It also signifies a shift wherein the sense of loss associated with the disability no longer holds significant influence in evaluating one’s self-worth. In other words, it can be interpreted as overcoming one’s own perception of the disability [12]. This concept of disability acceptance is typically composed of four main domains: an attitude of recognizing and respecting various values in life beyond the disability; a focus on internal resources (e.g., personality, abilities) over physical attributes; a perspective that limits the disability’s influence rather than allowing it to dominate one’s entire identity; and a shift in values to concentrate on one’s own assets and strengths rather than comparing oneself to others [13].

A more positive acceptance of one’s disability reduces negative self-perceptions, fosters self-esteem, and increases confidence in interpersonal relationships and the motivation for social participation, which in turn leads to an improved quality of life [14]. Conversely, a negative perception or rejection of one’s disability can trigger low self-esteem, social withdrawal, and negative emotional experiences, which can easily lead to depression [15]. Numerous previous studies [14,16] have revealed that the degree of disability acceptance is closely related to self-esteem, social participation, competence, and life satisfaction, while a lower degree of acceptance is linked to increased depression, anxiety, and stress [17]. This is further supported by research showing that if the acceptance process is not properly established in the early stages after acquiring a disability, psychological distress such as post-traumatic stress can persist and elevate the risk of developing depression [18]. Disability acceptance is influenced not only by an individual’s disability-related characteristics and attitudes, such as the type, age of onset, and severity of the disability, but also by social factors, necessitating a more multidimensional approach [19].

Although disability acceptance shares conceptual similarities with related constructs such as resilience, post-traumatic growth, and acceptance-based therapeutic frameworks (e.g., Acceptance and Commitment Therapy), it represents a distinct psychosocial process [20]. Resilience generally refers to adaptive functioning in response to adversity, while post-traumatic growth emphasizes positive psychological change following traumatic experiences [21]. In contrast, disability acceptance specifically involves the integration of disability into one’s self-identity and a reorientation of personal values, whereby disability is no longer perceived as the defining core of the self [22]. Acceptance-based therapies aim to enhance psychological flexibility within clinical contexts, whereas disability acceptance reflects a broader, lived psychosocial process shaped by social participation, stigma, and structural conditions beyond therapeutic settings [23].

However, existing research has primarily focused on the relationship between disability acceptance and positive psychological outcomes [14,15,16], with a lack of studies directly investigating the link between the degree of disability acceptance and the experience of depression. This study aims to fill this gap by empirically analyzing the relationship between the level of disability acceptance and the experience of depression among individuals with disabilities. By doing so, it seeks to move beyond reducing the mental health of people with disabilities to a mere pathological issue and instead explore an integrated, preventive approach centered on psychosocial factors. Furthermore, the findings of this study can inform the direction of policy interventions and serve as foundational data for developing counseling and social programs aimed at promoting disability acceptance.

Accordingly, the primary objective of this study is to examine disability acceptance as an independent psychosocial factor associated with depressive experience among people with disabilities. Although related constructs such as self-esteem and social participation are discussed in prior literature, this study does not aim to formally test mediation or moderation mechanisms. Rather, it focuses on establishing a robust population-based association that may serve as a foundation for future longitudinal and mechanistic research.

## 2. Materials and Methods

### 2.1. Study Design and Research Data

This study employed a cross-sectional analytical design using data from the second wave (2016–2018) of the Panel Survey of Employment for the Disabled (PSED), conducted by the Ministry of Employment and Labor and the Korea Employment Agency for Persons with Disabilities. The PSED is a nationally representative longitudinal panel survey initiated in 2008 to monitor employment status, social participation, and quality of life among registered people with disabilities in Korea.

Although the PSED is a longitudinal survey, the present analysis was restricted to a single survey wave. Disability acceptance and depressive experience were therefore assessed concurrently, and the temporal sequence between exposure and outcome could not be determined. Accordingly, the findings should be interpreted as associative rather than causal.

Of the 4577 registered individuals with disabilities aged 15–64 who participated in Wave 2, 4030 participants were included in the final analytic sample. Respondents were excluded if they did not provide complete responses to all 12 items of the disability acceptance scale or if data were missing for key covariates included in the multivariable models. Data were collected through face-to-face interviews using Tablet PC–Assisted Personal Interviewing (TAPI), which enhances data quality through automated question routing and real-time error checks.

### 2.2. Dependent Variable

The dependent variable was the experience of depression. This measure reflects self-perceived depressive experience rather than clinically diagnosed depression, as the PSED does not include validated psychiatric screening instruments or physician-diagnosed depression variables. The response (Yes/No) to the single question, “Have you experienced depression in the past year?” was used. This defined the respondent’s experience of depression as a dichotomous variable.

### 2.3. Independent Variable

The main independent variable was the degree of disability acceptance, measured using a 12-item scale (e.g., “I am satisfied with my life even though I have a disability,” “How you live your life is more important than the disability itself”) on a 5-point Likert scale (1 = Strongly disagree, 5 = Strongly agree). The scores for each item were summed to calculate a total score (ranging from 12 to 60), with higher scores indicating a higher level of disability acceptance. For analysis, scores were categorized into five groups for comparison: 20 or below, 21–30, 31–40, 41–50, and 51 or above.

This scale captures multiple dimensions of disability acceptance, including value reorientation, identity integration, and perceived control over life beyond physical limitations. Higher scores indicate a greater degree of psychosocial adaptation rather than disability severity or functional impairment.

Although the PSED does not provide formal psychometric validation indices within the publicly available dataset, the disability acceptance scale is grounded in well-established theoretical frameworks that conceptualize acceptance as a multidimensional psychosocial construct. Similar acceptance-based measures have demonstrated consistent associations with mental health outcomes in prior studies. In the present study, the observed dose–response relationship and robustness across multiple model specifications support the construct validity of this measure in the study population.

### 2.4. Control Variables

Covariates were selected based on prior literature on depression among people with disabilities and data availability in the PSED. Sociodemographic variables included gender, age group, marital status, residential region, and smoking status. Socioeconomic characteristics were assessed using economic activity status. Disability-related characteristics were adjusted for using type of disability, categorized as mobility/brain lesion disabilities versus other disability types, consistent with previous population-based studies.

Gender was classified as ‘female’ or ‘male’. Age was categorized as ‘15–29’, ‘30–39’, ‘40–49’, ‘50–59’, and ‘60–66’. Region of residence was divided into ‘Metropolitan area’, ‘Major city area’, and ‘Other cities/provinces’. Marital status was grouped into ‘Married/cohabiting’, ‘Single’, and ‘Divorced/widowed/separated’. Disability type was categorized as ‘Mobility/Brain lesion’ and ‘Other’. Economic activity status was divided into ‘Wage worker’, ‘Self-employed’, ‘Unpaid family worker’, ‘Unemployed’, and ‘Economically inactive population’. Health behavior factors included ‘smoking experience’. Smoking status was classified as ‘Non-smoker’, ‘Smoker’, and ‘Former smoker’.

Disability grade was initially considered as a potential covariate; however, it was excluded from the final models due to conceptual overlap and multicollinearity concerns with the main exposure variable (disability acceptance) and disability type. Including disability grade alongside these variables risked over-adjustment and instability of model estimates. All selected covariates were entered simultaneously into the multivariable logistic regression models.

### 2.5. Analysis Method

This study first examined the general characteristics of the subjects and the distribution of depression experience using descriptive statistics. Next, a chi-square test was conducted to verify the association between the degree of disability acceptance and the experience of depression. Furthermore, after controlling for sociodemographic and health behavior factors, a logistic regression analysis was performed to estimate the effect of disability acceptance on the experience of depression. The results are presented as odds ratios (OR) with 95% confidence intervals (95% CI). Prior to model estimation, Multicollinearity among covariates was assessed using variance inflation factors (VIFs), with all values below 2.5. Model discrimination was evaluated using the C-statistic (equivalent to the area under the ROC curve), and overall model fit was assessed using likelihood ratio tests and information criteria. Disability acceptance was analyzed using both categorical and continuous specifications to evaluate functional form robustness and potential dose–response relationships. This study was not designed to perform formal psychometric validation of the disability acceptance scale; rather, it aimed to examine the population-level association between theoretically grounded acceptance constructs and depressive experience. All statistical analyses were conducted using SAS 9.4.

## 3. Results

Among the 4030 study participants, 18.0% (n = 724) reported experiencing depression within the past year. The prevalence of depression showed a clear difference according to the degree of disability acceptance. In the group with the lowest acceptance scores (20 points or less), the depression experience rate was the highest at 59.7%. Conversely, in the group with the highest scores (51–60 points), the rate was the lowest at 5.1%. This clearly demonstrates an inverse dose–response relationship, where a higher level of disability acceptance is associated with a lower rate of depression (Table 1).

Table 2 presents the results of the logistic regression analysis, which examined the association between the degree of disability acceptance and the experience of depression after controlling for covariates. Compared to the reference group (51–60 points), the group with 20 points or less was approximately 11.4 times more likely to experience depression (OR = 11.353, 95% CI: 5.859–21.999, *p* < 0.0001). The 21–30 point group was 5.7 times more likely (OR = 5.721, 95% CI: 3.208–10.203, *p* < 0.0001), and the 31–40 point group was 2.3 times more likely (OR = 2.342, 95% CI: 1.323–4.147, *p* = 0.004). The 41–50 point group was also 1.36 times more likely (OR = 1.362, 95% CI: 0.762–2.435), but this finding was not statistically significant (*p* = 0.297). Model discrimination was acceptable, with C-statistic ranging from 0.75 to 0.76 across the main and sensitivity models. These findings indicate that disability acceptance is a robust correlate of depressive experience across multiple model specifications. The multivariable logistic regression model demonstrated good overall fit (likelihood ratio χ^2^ = 434.9, *p* < 0.0001) and acceptable discrimination, with a C-statistic of 0.75.

To assess the robustness of the main findings, several supplementary analyses were conducted. First, the association between disability acceptance and depressive experience was re-examined using an alternative four-category classification of disability type, and the results remained consistent with the primary model (Supplementary Table S1). Second, an interaction analysis between disability acceptance and disability severity was performed to evaluate potential effect modification by severity; no statistically significant interaction was observed (Supplementary Table S2). Disability severity was examined only in the interaction analysis and was not included as a covariate in the main models due to conceptual overlap with disability acceptance and disability type. Finally, disability acceptance was modeled as a continuous variable, and a consistent inverse association with depressive experience was observed, supporting the dose–response relationship identified in the categorical analyses (Supplementary Table S3). The consistency of effect estimates across categorical, continuous, and sensitivity analyses further supports the internal coherence and construct validity of the disability acceptance measure used in this study.

## 4. Discussion

This study empirically analyzed the relationship between the level of disability acceptance and the experience of depression using data from the nationally representative Panel Survey of Employment for the Disabled. The results clearly showed that a lower level of disability acceptance was associated with a significantly higher likelihood of experiencing depression, a trend that persisted even after controlling for sociodemographic and health behavior factors. This indicates that disability acceptance is not merely an adaptive process but a key protective factor for the mental health of individuals with disabilities.

The association between disability acceptance and depressive experience may operate through multiple psychosocial pathways, including reduced internalized stigma, enhanced coping capacity, and greater social participation [24]. However, due to the cross-sectional design, causal direction cannot be established, and bidirectional relationships are plausible.

These findings suggest that the mental health of individuals with disabilities is not solely determined by medical factors but is greatly influenced by the individual’s process of disability acceptance, in addition to external stressors such as social stigma, isolation, and economic constraints. In other words, positive acceptance of a disability strengthens self-esteem and improves quality of life, whereas a lack of acceptance can intensify negative self-perceptions, leading to depression [14]. This study is significant in that it expands upon previous research reporting a positive correlation between disability acceptance and quality of life/social participation [14,15,16], by empirically confirming that disability acceptance can also play a direct role in preventing depression.

It has already been reported in studies including various disability types that the prevalence of depression among people with disabilities is higher than in the general population worldwide [25,26]. In line with this trend, our study sought to understand the mechanisms of depression development in individuals with disabilities more precisely by highlighting disability acceptance as a key mental health variable. While previous studies [14,15,16] primarily focused on positive aspects like self-esteem and quality of life in relation to the degree of disability acceptance, our study holds significant importance by confirming the impact of acceptance levels on negative mental health outcomes such as depression, thereby providing foundational data for improving the mental health and quality of life for people with disabilities.

The magnitude of association observed in this study appears stronger than that reported in several studies from other high-income countries, suggesting that contextual factors such as social stigma and limited community-based mental health resources in Korea may amplify the mental health consequences of low disability acceptance.

Disability acceptance involves more than simply acknowledging the disability; it includes a psychological process of reconstruction that internally integrates the life changes brought about by the disability [27]. This process involves repositioning the disability as a part of life rather than its center and adjusting its significance within one’s self-identity [27]. Individuals with disabilities form their identity through social, emotional, and behavioral development processes that go beyond mere self-perception [28]. This development is generally specified in four stages: first, accepting one’s disability (acceptance); then, forming relationships with other people with disabilities (relationship); adopting the disability as part of one’s identity (adoption); and finally, practicing social participation and advocating for rights as a person with a disability (engagement) [28]. In other words, disability acceptance can be described as a complex and dynamic psychosocial process of reconstructing one’s identity and life’s meaning.

Furthermore, disability acceptance involves a shift in the meaning attributed to the disability. What was initially perceived as loss, frustration, or shame may be reinterpreted as an opportunity for growth or a chance to explore new values and directions as acceptance progresses [29]. From a post-traumatic growth perspective, a physical disability can become a catalyst for re-evaluating life’s meaning and priorities [30], and this process leads to well-being, including personal growth, a sense of control over one’s life, and restored confidence in interpersonal relationships [29].

Most importantly, this acceptance process does not follow a fixed, single path; it unfolds in various ways depending on the individual’s disability type, time of onset, social support network, and pre-existing self-concept [27,28,29,30]. If the disability acceptance process does not proceed smoothly, it can lead to a failure in assigning integrated meaning to life and intensify negative self-perceptions, resulting in mental health problems like depression.

Depression is influenced not only by internal factors like disability acceptance but also by structural factors such as social support, social activities, and employment retention. Economic participation enables financial independence for people with disabilities and positively affects their physical and mental health [31,32]. According to a prior study on the health impacts of changes in employment status for people with disabilities [33], groups with no work experience or who became unemployed had lower health levels than those who maintained continuous economic activity. The fact that stable employment and social participation reduce the risk of depression has been repeatedly confirmed [34,35]. Disability acceptance also interacts with these social and structural factors, shaping its manifestation [36]. Social exclusion, perceived discrimination [36], job loss, and economic difficulties [19] hinder disability acceptance, which in turn leads to increased depression, anxiety, and stress. This suggests that disability acceptance should be understood not as a purely internal issue, but as a relational psychological state that is heavily dependent on the presence of social infrastructure and environmental resources.

In addition, people with disabilities are affected not only by external stigma but also by ‘internalized ableism,’ where they internalize negative societal perceptions about disability [37]. This leads to decreased self-efficacy [38], identity confusion, and low self-esteem, thereby increasing the likelihood of developing depression [39]. The stronger the internalized ableism, the greater the resistance to accepting the disability as part of the self, which can lead to lower levels of disability acceptance along with depression, helplessness, and isolation.

In conclusion, this study empirically demonstrates that disability acceptance plays a pivotal role in the mental health of individuals with disabilities. This implies the need for a multi-layered approach that combines not just individual psychological interventions but also social solidarity, employment and community support, and educational and cultural changes to eliminate stigma. When a social environment that promotes disability acceptance is established, individuals with disabilities will be better able to healthily integrate their identity, reduce their risk of depression and suicide, and maintain a psychosocially stable life.

Interventions aimed at enhancing disability acceptance may be most feasible when integrated into existing disability employment services and community mental health systems in Korea, where regular contact with people with disabilities already occurs.

### Limitations

This study has several limitations. First, because it uses cross-sectional survey data, it is difficult to establish clear causal relationships between disability acceptance and depressive experience. Second, as all variables were assessed using self-reported questionnaires, the findings may be subject to reporting bias. Third, the analytic sample consisted of registered persons with disabilities in Korea, which may limit the generalizability of the results to the broader population of people with disabilities, and selective attrition across survey waves may have further affected representativeness. Nevertheless, this study is meaningful in that it empirically identified a robust association between disability acceptance and depressive experience using nationally representative data. Although disability acceptance and depression were not measured using clinically validated diagnostic instruments, the primary aim of this study was not psychometric validation or scale development, but rather to establish a population-based association between a theoretically grounded psychosocial construct and depressive experience.

## 5. Conclusions

In conclusion, this study confirmed that disability acceptance is a key factor not just for adaptation but for the emotional stability and depression prevention of individuals with disabilities. These findings provide crucial evidence for developing interventions and policies aimed at promoting mental health and social integration. Moving forward, a public health strategy is needed to reduce depression and suicide risk among people with disabilities by promoting disability acceptance through a multidimensional approach that encompasses counseling, education, employment support, and stigma reduction.

## Figures and Tables

**Table 1:** The general characteristics of the participants included in the analysis.

Characteristics	TOTAL N (%)	Depression: Yes N (%)	Depression: No N (%)	*p*-Value
Level of disability acceptance *				<0.0001
≤20	62 (1.54)	37 (59.7)	25 (40.3)	
21–30	603 (14.96)	203 (33.7)	400 (66.3)	
31–40	2212 (54.89)	373 (16.9)	1839 (83.1)	
41–50	1054 (26.15)	106 (10.1)	948 (89.9)	
51–60	99 (2.46)	5 (5.1)	94 (95.0)	
Gender				0.004
Male	2626 (65.16)	438 (16.7)	2188 (83.3)	
Female	1404 (34.84)	286 (20.4)	1118 (79.6)	
Age (years)				<0.0001
15–29	699 (17.34)	95 (13.6)	604 (86.4)	
30–39	975 (24.19)	149 (15.3)	826 (84.7)	
40–49	1164 (28.88)	216 (18.6)	948 (81.4)	
50–59	781 (19.38)	181 (23.2)	600 (76.8)	
60–66	411 (10.20)	83 (20.2)	328 (79.8)	
Marital status				<0.0001
Married/Cohabiting	1806 (44.81)	220 (12.2)	1586 (87.8)	
Never married	1624 (40.30)	310 (19.1)	1314 (80.9)	
Divorced/Widowed/Separated	600 (14.89)	194 (32.3)	406 (67.7)	
Residential region				0.189
Capital area	782 (19.40)	149 (19.1)	633 (81.0)	
Metropolitan cities	1168 (28.98)	190 (16.3)	978 (83.7)	
Other provinces	2080 (51.61)	385 (18.5)	1695 (81.5)	
Smoking status				0.007
Current smoker	917 (22.75)	195 (21.3)	722 (78.7)	
Former smoker	762 (18.91)	139 (18.2)	623 (81.8)	
Never smoked	2351 (58.34)	390 (16.6)	1961 (83.4)	
Economic activity status				<0.0001
Wage worker	1550 (38.46)	167 (10.8)	1383 (89.2)	
Self-employed	395 (9.80)	40 (10.1)	355 (89.9)	
Unpaid family worker	61 (1.51)	8 (13.1)	53 (86.9)	
Unemployed	144 (3.57)	35 (24.3)	109 (75.7)	
Economically inactive	1880 (46.65)	474 (25.2)	1406 (74.8)	
Type of disability				0.002
Mobility/Brain lesion	2000 (49.63)	321 (16.1)	1679 (84.0)	
Others	2030 (50.37)	403 (19.9)	1627 (80.2)	
Total	4030 (100.0)	724 (18.0)	3306 (82.0)	

* Higher scores indicate higher levels of disability acceptance.

**Table 2:** Association between disability acceptance and depressive experience among people with disabilities.

Variable	Category	Odds Ratio (OR) *	95% CI	*p*-Value
Level of disability acceptance ^†^	41–50	1.14	0.40–3.24	0.813
	31–40	2.58	0.92–7.19	0.070
	21–30	5.81	2.06–16.36	<0.001
	Highest (51–60)	1.00	Reference	–
	Lowest (≤20)	4.96	1.60–15.40	0.006
Gender	Female	1.00	Reference	–
	Male	0.72	0.57–0.91	0.007
Age (years)	60–66	1.00	Reference	–
	15–29	0.75	0.50–1.13	0.169
	30–39	1.29	0.91–1.81	0.154
	40–49	1.00	0.73–1.35	0.972
	50–59	1.37	1.02–1.83	0.034
Marital status	Never married	1.00	Reference	–
	Divorced/Widowed/Separated	1.35	1.01–1.80	0.040
	Married/Cohabiting	0.68	0.53–0.88	0.003
Residential region	Other provinces	1.00	Reference	–
	Capital area	1.53	1.23–1.90	<0.001
	Metropolitan cities	0.94	0.72–1.22	0.633
Smoking status	Never smoked	1.00	Reference	–
	Former smoker	1.24	0.94–1.65	0.133
	Current smoker	1.32	1.00–1.73	0.050
Economic activity ^‡^	Type 5 ^†^	1.00	Reference	–
	Type 1	0.34	0.27–0.44	<0.001
	Type 2	0.49	0.33–0.71	<0.001
	Type 3	0.27	0.08–0.89	0.031
	Type 4	1.03	0.61–1.75	0.912
Type of disability	Others	1.00	Reference	–
	Mobility/Brain lesion	1.21	1.00–1.46	0.057
Survey year	2018	1.00	Reference	–
	2016	1.19	0.83–1.71	0.355
	2017	1.21	0.88–1.68	0.244

* OR = odds ratio; CI = confidence interval. C-statistic (c): 0.751. ^†^ Disability acceptance was reverse-coded, such that higher category values indicate lower levels of acceptance. ^‡^ Economic activity categories were defined according to the Panel Survey of Employment for the Disabled (PSED).

## Data Availability

The data used in this study are available from the Korea Employment Agency for Persons with Disabilities, but restrictions apply to their availability. Data are available from the authors upon reasonable request and with permission of the data provider.

## Supplementary Materials

The following supporting information can be downloaded at: https://www.mdpi.com/article/10.3390/medicina62020301/s1, Table S1: Sensitivity analysis of the association between disability acceptance and depressive experience using a four-category classification of disability type; Table S2: Interaction between disability acceptance and disability severity in relation to depressive experience; Table S3: Association between continuous disability acceptance score and depressive experience.

## References

[1] Chang S.M., Hahm B.-J., Lee J.-Y., Shin M.S., Jeon H.J., Hong J.-P., Lee H.B., Lee D.-W., Cho M.J. (2008). Cross-national difference in the prevalence of depression caused by the diagnostic threshold. J. Affect. Disord..

[2] Kim A.M. (2020). Factors associated with the suicide rates in Korea. Psychiatry Res..

[3] Azeem B., Siddiqui M.R., Shaikh S., Sattar A., Saeed H. (2025). Depression leading to suicide in United States: A retrospective analysis of CDC WONDER from 1999 to 2022. J. Psychiatr. Res..

[4] Melchior M., Florence A.-M., Davisse-Paturet C., Falissard B., Galéra C., Hazo J.-B., Vuillermoz C., Warszawski J., Dione F., Rouquette A. (2022). Labor market participation and depression during the COVID-19 pandemic among young adults (18 to 30 years): A nationally representative study in France. Front. Public Health.

[5] Reddy M. (2010). Depression: The Disorder and the Burden.

[6] Shen S.-C., Huang K.-H., Kung P.-T., Chiu L.-T., Tsai W.-C. (2017). Incidence, risk, and associated factors of depression in adults with physical and sensory disabilities: A nationwide population-based study. PLoS ONE.

[7] Park G.-R., Haseeb S., Namkung E.H. (2025). The effects of poor housing conditions on depressive symptoms in persons with disabilities: Do neighborhood resources and residence type matter?. Disabil. Health J..

[8] Hsieh N., Waite L. (2019). Disability, psychological well-being, and social interaction in later life in China. Res. Aging.

[9] Nagata S., McCormick B., Brusilovskiy E., Salzer M.S. (2022). Community participation as a predictor of depressive symptoms among individuals with serious mental illnesses. Int. J. Soc. Psychiatry.

[10] Ogawa M., Fujikawa M., Jin K., Kakisaka Y., Ueno T., Nakasato N. (2021). Acceptance of disability predicts quality of life in patients with epilepsy. Epilepsy Behav..

[11] Lim M.J.R., Tan J., Neo A.Y.Y., Ng B.C.J., Asano M. (2024). Acceptance of disability in stroke: A systematic review. Ann. Phys. Rehabil. Med..

[12] Byra S., Żyta A. Acceptance of disability, self-efficacy and hope for success in students with physical disability. Proceedings of the 3rd International Conference on Lifelong Education and Leadership for All Iclel 2017.

[13] Forber-Pratt A.J., Merrin G.J., Mueller C.O., Price L.R., Kettrey H.H. (2020). Initial factor exploration of disability identity. Rehabil. Psychol..

[14] Kim H.m. (2023). Effects of self-efficacy, self-esteem, and disability acceptance on the social participation of people with physical disabilities: Focusing on COVID-19 pandemic. Brain Behav..

[15] Jung Y.H., Kang S.H., Park E.-C., Jang S.-Y. (2022). Impact of the acceptance of disability on self-esteem among adults with disabilities: A four-year follow-up study. Int. J. Environ. Res. Public Health.

[16] Chen R.K., Crewe N.M. (2009). Life satisfaction among people with progressive disabilities. J. Rehabil..

[17] Han A., Lee J., Kim J., Choi H., Ju H.-J. (2025). Longitudinal analysis of disability acceptance and disability-related stress in people with intellectual and developmental disabilities. J. Intellect. Dev. Disabil..

[18] O’Donnell M.L., Holmes A.C., Creamer M.C., Ellen S., Judson R., McFarlane A.C., Silove D.M., Bryant R.A. (2009). The role of post-traumatic stress disorder and depression in predicting disability after injury. Med. J. Aust..

[19] Park E.-Y., Kim J.-H. (2021). Interaction of socio-demographic characteristics on acceptance of disability among individuals with physical disabilities. Front. Psychiatry.

[20] Kim E., Park J. (2023). Structural Relationship among Disability Acceptance, Resilience, and Social Participation of Adolescents with Physical Disabilities. Int. J. Disabil. Dev. Educ..

[21] Infurna F.J., Jayawickreme E., Woods-Jaeger B., Zalta A.K. (2024). Understanding adaptive responses to adversity: Introduction to the special issue on rethinking resilience and posttraumatic growth. Am. Psychol..

[22] Botha S.C., Harvey C. (2024). Doing difference differently: Identity (re) constructions of adults with acquired disabilities. Rehabil. Psychol..

[23] Bonacquisti A., Woodworth E.C., Diaz M., Grunberg V.A. (2024). Acceptance-based process variables on postpartum well-being and distress: The mediating role of psychological flexibility. PLoS ONE.

[24] Montesano C., Liberali G., Azzali G., Buzzachera C., Angilletta S., Alessandria M., Guidetti L., De Giorgio A. (2025). Reducing Stigma, Enhancing Psychological Well-Being and Identity in Multiple Sclerosis: A Narrative Review of Current Practices and Future Directions. Healthcare.

[25] Asdaq S.M.B., Alshehri S., Alajlan S.A., Almutiri A.A., Alanazi A.K.R. (2024). Depression in persons with disabilities: A scoping review. Front. Public Health.

[26] Okoro C.A., Dhingra S.S. (2014). Severity of psychological distress among adults with and without disabilities. Soc. Work Public Health.

[27] Adler J.M., Lakmazaheri A., O’Brien E., Palmer A., Reid M., Tawes E. (2021). Identity integration in people with acquired disabilities: A qualitative study. J. Personal..

[28] Forber-Pratt A.J., Zape M.P. (2017). Disability identity development model: Voices from the ADA-generation. Disabil. Health J..

[29] Szcześniak M., Świątek A.H., Cieślak M., Świdurska D. (2020). Disease acceptance and eudemonic well-being among adults with physical disabilities: The mediator effect of meaning in life. Front. Psychol..

[30] Elliott T.R., Kurylo M., Rivera P. (2002). Positive growth following acquired physical disability. Handbook of Positive Psychology.

[31] Saxby K., Dickinson H., Petrie D., Kavanagh A., Aitken Z. (2023). The impact of employment on mental healthcare use among people with disability: Distinguishing between part-and full-time employment. Scand. J. Work Environ. Health.

[32] Lee J., Lee E. (2010). The relationship of participation in leisure sports on stress and the decrease of suicidal thought of elderly. J. Leis. Recreat. Stud..

[33] Barišin A., Benjak T., Vuletić G. (2011). Health-related quality of life of women with disabilities in relation to their employment status. Croat. Med. J..

[34] Jung S.W., Yoon J.-H., Lee W. (2021). Predictors for depressive symptoms by four types of disability. Sci. Rep..

[35] Li F., Wang S., Gao Z., Qing M., Pan S., Liu Y., Hu C.J.F.i.M. (2025). Harnessing artificial intelligence in sepsis care: Advances in early detection, personalized treatment, and real-time monitoring. Front. Med..

[36] Li L., Moore D. (1998). Acceptance of disability and its correlates. J. Soc. Psychol..

[37] Jóhannsdóttir Á., Egilson S.Þ., Haraldsdóttir F. (2022). Implications of internalised ableism for the health and wellbeing of disabled young people. Sociol. Health Illn..

[38] Pearl R.L., Li Y., Groshon L.C., Hernandez M., Saunders D., Sheynblyum M., Driscoll K.A., Gelfand J.M., Manavalan P., Montanez-Wiscovich M. (2024). Measuring internalized health-related stigma across health conditions: Development and validation of the I-HEARTS Scale. BMC Med..

[39] O’Donnell A.T., Foran A.-M. (2024). The link between anticipated and internalized stigma and depression: A systematic review. Soc. Sci. Med..

