# Supplementary material for: Disability Acceptance as a Key Protective Factor Against Depression: Evidence from Korea’s National PSED (Wave 2)
Source: Medicina (Kaunas). 2026 Feb 2;62(2):301. doi: 10.3390/medicina62020301 (PMC12942481; doi:10.3390/medicina62020301)
Supplement: Supplementary file 1 [file medicina-62-00301-s001.zip › medicina-4074668-supplementary.pdf]

Supplementary Table S1. Sensitivity analysis of the association between disability acceptance and depressive experience using a four-category classification of disability type.

Variable	Category	Odds Ratio (OR)	95% CI	p-value
Level of disability acceptance*	Lowest (≤20)	5.29	1.70–16.46	0.004
	41–50	1.18	0.41–3.39	0.753
	31–40	2.68	0.96–7.48	0.061
	21–30	6.01	2.13–16.97	<0.001
	Highest (51–60)	1.00	Reference	–
Gender	Female	1.00	Reference	–
	Male	0.72	0.57–0.92	0.008
Age (years)	60–66	1.00	Reference	–
	15–29	0.75	0.50–1.12	0.161
	30–39	1.27	0.90–1.80	0.172
	40–49	1.01	0.74–1.37	0.962
	50–59	1.38	1.03–1.84	0.031
Marital status	Never married	1.00	Reference	–
	Divorced/Widowed/Separated	1.33	0.99–1.77	0.057
	Married/Cohabiting	0.67	0.51–0.87	0.002
Residential region	Other provinces	1.00	Reference	–
	Capital area	1.52	1.22–1.88	<0.001
	Metropolitan cities	0.93	0.72–1.22	0.611
Smoking status	Never smoked	1.00	Reference	–
	Former smoker	1.22	0.92–1.62	0.174
	Current smoker	1.31	1.00–1.73	0.054
Economic activity	Type 5†	1.00	Reference	–
	Type 1	0.34	0.27–0.43	<0.001
	Type 2	0.48	0.32–0.70	<0.001
	Type 3	0.27	0.08–0.89	0.031
	Type 4	1.03	0.61–1.76	0.904
Type of disability	Sensory/Communication	1.00	Reference	–
	Internal organ/Other	1.82	1.17–2.83	0.008
	Mobility/Brain lesion	1.23	0.98–1.56	0.080
	Neurodevelopmental/Mental	0.89	0.65–1.21	0.446
Survey year	2018	1.00	Reference	–
	2016	1.18	0.82–1.70	0.369
	2017	1.21	0.87–1.67	0.256

* OR = odds ratio; CI = confidence interval. C-statistic (c): 0.754.

† Disability acceptance was reverse-coded, such that higher category values indicate lower levels of acceptance.

‡ Economic activity categories were defined according to the Panel Survey of Employment for the Disabled (PSED).

Supplementary Table S2. Interaction between disability acceptance and disability severity in relation to depressive experience.

Variable	Category	Odds Ratio (OR)	95% CI	p-value
Level of disability acceptance*	Reference group	–	–	–
Disability severity	Grades 1–3 (Severe)	1.00	Reference	–
	Grades 4–6 (Mild)	0.30	0.03–2.66	0.256
Acceptance × Severity	acc_ref 2 × Mild	3.05	0.36–25.6	0.311
	acc_ref 3 × Mild	2.50	0.32–19.4	0.390
	acc_ref 4 × Mild	5.09	0.73–35.7	0.131

Variable	Category	Odds Ratio (OR)	95% CI	p-value
	acc_ref 5 × Mild	6.01	0.74–48.9	0.132
Covariates	Same as Table 2	Adjusted	–	–

* OR = odds ratio; CI = confidence interval. C-statistic (c): 0.754.

Interaction terms between disability acceptance level and disability severity were included in multivariable logistic regression models.

No statistically significant interaction was observed, indicating that the association between disability acceptance and depressive experience did not differ by disability severity.

Models were adjusted for the same covariates as in Table 2.

Supplementary Table S3. Association between continuous disability acceptance score and depressive experience.

Variable	Category	Odds Ratio (OR)	95% CI	p-value
Disability acceptance score	Per 1-point increase	0.93	0.92–0.95	<0.001
Gender	Female	1.00	Reference	–
	Male	0.74	0.59–0.94	0.013
Age (years)	60–66	1.00	Reference	–
	15–29	0.77	0.52–1.16	0.209
	30–39	1.27	0.90–1.79	0.173
	40–49	0.98	0.72–1.33	0.873
	50–59	1.37	1.03–1.83	0.031
Marital status	Never married	1.00	Reference	–
	Divorced/Widowed/Separated	1.42	1.06–1.89	0.017
	Married/Cohabiting	0.71	0.55–0.92	0.010
Residential region	Other provinces	1.00	Reference	–
	Capital area	1.55	1.25–1.92	<0.001
	Metropolitan cities	0.95	0.73–1.23	0.690
Smoking status	Never smoked	1.00	Reference	–
	Former smoker	1.21	0.91–1.60	0.194
	Current smoker	1.29	0.98–1.70	0.065
Economic activity	Type 5†	1.00	Reference	–
	Type 1	0.34	0.27–0.44	<0.001
	Type 2	0.47	0.32–0.69	<0.001
	Type 3	0.27	0.08–0.87	0.029
	Type 4	1.07	0.63–1.80	0.814
Type of disability	Others	1.00	Reference	–
	Mobility/Brain lesion	1.24	1.02–1.50	0.028

* OR = odds ratio; CI = confidence interval. C-statistic (c): 0.754.

Odds ratios represent the change in odds of depressive experience per one-point increase in disability acceptance score.

Models were adjusted for the same covariates as in Table 2.
